# Workplace Vaccination Against COVID-19 and Seasonal Influenza in the United States: A Modeling-Based Estimation of the Health and Economic Benefits for Employers and Employees

**DOI:** 10.3390/jmahp13020017

**Published:** 2025-04-24

**Authors:** Ekkehard Beck, Keya Joshi, Darshan Mehta, Stephane Lorenc, Bishoy Rizkalla, Nicolas Van de Velde

**Affiliations:** 1 Moderna, Inc., 325 Binney St, Cambridge, MA 02142, USA; 2 EREVAS Sàrl, 29 Boulevard du Prince Henri, Luxembourg L-1724, Luxembourg

**Keywords:** COVID-19, influenza, vaccination, workplace, economic model, United States

## Abstract

The objectives were to assess the economic burden of COVID-19 and impact of workplace COVID-19 vaccination in the United States (US). An economic model estimated COVID-19 workplace burden (infections, long COVID, inpatient/outpatient care, absent days) with and without vaccination, compared with seasonal influenza vaccination for context, using Optum’s de-identified Clinformatics^®^ Data Mart Database. Without workplace vaccination, an average US business (with 10,000 employees), had 18,175 absent days from COVID-19 and lost productivity costs of USD 5.08 million. Implementing COVID-19 workplace vaccination (at 70% coverage) prevented approximately 3132 absent days, saving employers USD 876,453 (lost productivity) and USD 240,633 (medical costs); and saving employees USD 182,196 (medical costs) and USD 198,250 (lost wages) versus no COVID-19 workplace vaccination. The burden and vaccination impact were greater for COVID-19 versus seasonal influenza. Workplace vaccination for COVID-19 and seasonal influenza can have a significant impact for both the employer and employees through averted disease.

## 1. Introduction

The coronavirus disease 2019 (COVID-19) pandemic had a major impact on the United States (US) economy, with a gross domestic product loss estimated at USD 14 trillion over the first four years of the pandemic (2020–2023) [1]. Industry experienced large declines in revenue (e.g., over 50% declines in the first 30 months for air travel, indoor dining, and large events [1]) as well as labor shortages due to reduced labor force participation [2].

According to the US Bureau of Labor Statistics, the number of employees absent due to illness remained elevated every quarter compared with pre-pandemic levels, especially during the winter months (e.g., 5.00 million and 3.30 million in the fourth quarter of 2020 and 2023, respectively, versus 2.85 million before the pandemic) [3]. For employees, the average loss of earnings due to a COVID-19 absence was estimated at USD 9000, with 90% of this loss occurring over the 14 months following infection [4]. For the overall US population, this amounted to an estimated annual loss in employee earnings due to COVID-19 of USD 62 billion, equivalent to around half of the loss from cancer or diabetes [4].

For employers, employee absences due to COVID-19 resulted in major disruptions to business operations. For instance, the surge in Omicron cases in late 2021/early 2022 caused absences among 4.4% of United Airlines employees and 10.7% of Delta employees, which resulted in up to 7% of flights being cancelled [5], compared with 1.6% of pre-pandemic flights cancelled in 2018 [6]. Outbreaks and transmission of infectious respiratory diseases are common in the workplace due to employee interactions. For example, in the second half of 2021, there were a reported 12,660 COVID-19 outbreaks with known type of workplace, of which nearly a third were in nursing/residential care facilities and nearly a quarter in K–12 (primary and secondary) schools [7]. By contrast, pooled estimates from seasonal influenza transmission studies indicate that 16.2% of influenza transmission occurs in the workplace [8]. The workplace impact of COVID-19 when compared with seasonal influenza may also lead to longer absences, as observed in a 2022 study from the Netherlands: there was a longer absence from work due to COVID-19 (median 10 days versus 6 for influenza), as well as a lower chance of returning to work in the short-term (34% vs. 72% within 1 week, 71% vs. 92% within 3 weeks, and 91% vs. 99% within 6 weeks) [9]. The impact of COVID-19-related absences is further exacerbated by an ongoing skills shortage in the US [10]. Many industries are experiencing difficulties in finding and retaining skilled workers, which magnifies the operational disruptions caused by employee illnesses. This shortage not only heightens the financial burden on businesses but also stresses the importance of maintaining a healthy workforce to sustain productivity and operations.

Following the acute infection and corresponding absences from work, long COVID can also result in long-term absences from work. In the US, a recent Centers for Disease Control and Prevention (CDC) study on long COVID reported that 5% of working-age adults with COVID-19 experienced long-term symptoms, of which 28% (and up to 35% of 40–49-year-olds) experienced disabling long COVID, significantly limiting their daily activities [11], and thus likely to impact their work productivity. In addition, claims data analyses of injured workers from New York State found that 18% of people with long COVID who filed an injury claim remained off work one year after contracting COVID-19, and the majority (78%) were aged <60 years [12]. The 2022 US National Health survey reported that people with long COVID have a nearly 30% higher chance of experiencing disability (odds ratio 1.29; 95% confidence interval [CI] 1.11–1.50) than those without long COVID [13]. In addition, 6% had chronic cardiovascular conditions and 39% had anxiety or depression resulting in an increased number of workdays missed versus people with those conditions but without long COVID (average 26.2 vs. 11.8 days for cardiovascular; and 12.2 vs. 8.5 for anxiety/depression) [13]. In 2021, the US government issued guidance that long COVID could be considered a disability under the American Disability Act (ADA), thus requiring employers to provide reasonable accommodations [14]. Total estimated annual losses in wages due to long COVID in the US were estimated to range from USD 105–235 billion [15,16].

COVID-19 remains the leading cause of respiratory infectious disease leading to hospitalizations and deaths, regardless of age and comorbidities, both during and between seasonal peaks [17]. To maintain high levels of protection against COVID-19, the CDC recommends a fall vaccination for all ages (from 6 months old) with an updated 2024–2025 COVID-19 vaccine. Seasonal influenza vaccination among working-age adults is relatively common, with May 2024 coverage rates of 37.5% among 18–49 year-olds and 51.5% among 50–64-year-olds in the US [18]; however, COVID-19 vaccination coverage was lower, 14.3% among 18–49-year-olds and 25.2% among 50–64-year-olds [19]. Seasonal influenza vaccination programs in the workplace are also common in the US, and previous studies have shown that annual, free, onsite seasonal influenza vaccination in the workplace was successful in achieving a pooled vaccination coverage rate of 42% (95% CI 25–60%) among employees [20]. Despite the low vaccination coverage rate among working-age adults and lack of data on COVID-19 workplace vaccination programs among employers in the US, available data indicate that workplace vaccination could be successful, as seen among healthcare workers [21].

To inform implementation of COVID-19 workplace vaccination programs, this analysis assessed the COVID-19 disease burden in the workplace and the impact of COVID-19 workplace vaccination with the updated Moderna COVID-19 mRNA Fall 2023/2024 vaccine. The impact was assessed for employers (e.g., productivity losses, medical costs) and employees (e.g., disease burden, wage losses and medical costs), and compared with scenarios of no workplace vaccination and seasonal influenza workplace vaccination. The aim was to help inform employers about how implementing a COVID-19 workplace vaccination program might compare to existing seasonal influenza workplace programs. A simplified decision tree economic model estimated disease burden of COVID-19 and seasonal influenza, and evaluated the health and economic outcomes following workplace vaccination among working-age adults in the US.

## 2. Materials and Methods

### 2.1. Model Description

**Figure 1:**
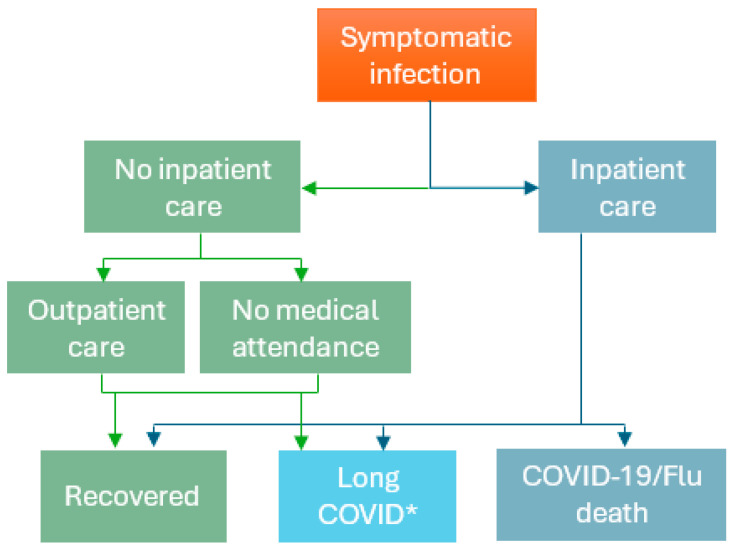
Workplace COVID-19 and seasonal influenza vaccination model structure *. * Only COVID-19 cases could develop long COVID; COVID-19: Coronavirus disease 2019; Flu: seasonal influenza; ICU: intensive care unit.

While dynamic transmission, static, or cost-effectiveness models could be used [22,23], a simplified static decision tree ‘workplace vaccination’ model (Figure 1) for both COVID-19 and seasonal influenza was selected, to provide meaningful insights into health and economic impacts relevant for employer decision-making. The model was developed in Microsoft Excel, based on the health and economic infection consequences part of a comprehensive COVID-19 dynamic transmission and economic model by Kohli et al. (2023) [24]. The workplace vaccination model was used to estimate the disease impact of COVID-19 and seasonal influenza (assuming no workplace vaccination in place), as well as the impact of Moderna COVID-19 mRNA Fall 2023/2024 vaccination and currently available seasonal influenza vaccination among employees aged 18–64 years over a one-year time horizon. Vaccination impact, i.e., the prevention of disease outcomes and associated medical costs, productivity, and wage losses in the vaccinated employees, was estimated based on COVID-19 and seasonal influenza disease-related health and economic outcomes prevented by vaccination, using current vaccination rates (based on the 2023–2024 season data) and the US Healthy People 2030 target immunization rate for seasonal influenza vaccination (70%) for both COVID-19 and seasonal influenza [25]. These results provide insights into the value for employers and employees of increasing vaccination rates in the working-age population.

Disease outcomes included infections, outpatient visits, hospitalizations, COVID-19 or seasonal influenza deaths, and long COVID (conservatively assuming only severe persistent long COVID impacts the ability to work and leads to absenteeism). Disease outcomes such as symptomatic infection with outpatient attendance, no outpatient attendance, and inpatient attendance were assumed to be mutually exclusive events. Economic outcomes included employer-specific outcomes such as workdays missed overall and due to long COVID (productivity loss was only due to absenteeism i.e., absence from the workplace. Presenteeism data, i.e., lower productivity from sick employees at the workplace, were not available);), direct medical costs (applicable for fully self-insured employers), and employee-specific outcomes such as employee co-pays for direct medical costs and wage losses for the employee due to only a limited number of sick days being covered by the employer. Considering the employer and employee perspective, productivity (employer) and wage (employee) losses and medical costs were included. Under the US Affordable Care Act (ACA), applicable large employers (ALEs) are required to offer healthcare coverage to their full-time employees and their dependents up to 26 years of age [26]. Thus, the model also included health outcomes and medical costs for employees’ dependents (children < 18 years), as well as productivity losses to the employer and wage losses to employees for time off to care for their dependents. The model focused on the impact of disease and vaccination in the workplace, thus, costs of COVID-19 and seasonal influenza vaccination and of potential vaccination-related adverse events were not included.

### 2.2. Model Structure

In Figure 1, employees/dependents with a symptomatic COVID-19 infection who are not hospitalized could either receive outpatient care or have no medical attendance, after which they can either recover or develop long COVID. For severe COVID-19 disease, employees/dependents may be hospitalized, after which they can recover, develop long COVID, or die due to COVID-19. The model did not include other causes of mortality over the 1-year time horizon as both COVID-19 and seasonal influenza were assumed to be annually recurring workplace vaccination programs. For seasonal influenza, the structure was similar; however, influenza cases were not assumed to develop long COVID, and conservatively also not assumed to develop cardiovascular events or exacerbations of other underlying chronic conditions [27]. The probabilities of these events, with their associated costs and numbers of workdays missed, are presented in Table S1. Vaccination was assumed to reduce the risk of symptomatic COVID-19 or seasonal influenza infection, as well as hospitalizations and their consequences among the vaccinated employees and dependents.

### 2.3. Model Inputs and Assumptions

Companies in the US are very heterogeneous with regards to size and many other aspects relevant for this study. To allow for some generalizability of results, a hypothetical US company was simulated considering average US data on employers, employees, and COVID-19 and seasonal influenza disease burden. Population averages were used due to a lack of company-based data. Extensive scenario and sensitivity analysis on input parameters related to the heterogeneity of companies in the US were conducted to provide further insights beyond the ‘average’ US company.

The base case analysis simulated a business with 10,000 employees and 0.86 dependents per employee [28], although results are presented for smaller and larger businesses as well (1000 and 100,000 employees) as results of the model followed a linear scale according to the number of employees. Overall, 60% of employees were assumed to be covered by health insurance [29], with a 25% employee co-pay, and up to 10 days of paid sick leave. Average US data were used for employee age distribution [30], annual wage (i.e., USD 61,442 [31]), and percentage currently vaccinated by age (e.g., 14.70–23.50% for COVID-19 [32] and 37.50–51.50% for seasonal influenza [18,33] among 18–64-year-olds).

In the model, without vaccination, 20.80% of employees developed symptomatic COVID-19 infections, assuming the Kohli et al. (2023) dynamic model’s projected incidence of COVID-19 during the 2023/2024 season (September 2023 to August 2024) in the counterfactual scenario (i.e., no update Fall 2023/2024 vaccination) (Table S2a) [24].

Then, 39.20% of employees with a symptomatic COVID-19 infection were assumed to incur outpatient visits (assumption based on seasonal influenza data [34]); 0.61% were hospitalized (based on analysis of non-vaccinated patients with any medical attendance from September 2023 to February 2024 using Optum’s de-identified Clinformatics^®^ Data Mart Database) and 2.07% of these died from COVID-19 [35]; and 2.2% developed severe long COVID resulting in workdays lost [11,36]. The impact on productivity loss was 3.57 days missed for a symptomatic infection [24], 42.31 days for hospitalization with subsequent recovery [24,37], and 82.40 days per year due to long COVID (see Supplemental File S1, Tables S3 and S4 for details) [12,38]. Taking a conservative approach, only the impact of COVID-19 on absenteeism (i.e., missed workdays) was considered, and not any potential impact on presenteeism i.e., working while being sick with reduced productivity and potential associated disability insurance payments. Associated medical costs (inflated to 2023 price levels [39]) were USD 477.17 [24] per employee with outpatient care, USD 22,853.01 [24] per hospitalization, and USD 517.32 [39,40] of post-COVID-19 cost assumed for each COVID-19 patient with any medical attendance. The cost to the employer of replacing an employee (because of COVID-19 death; no replacement due to long-COVID was assumed) was USD 14,724, based on a replacement duration of 56 days [41]. In addition, the model assumed 56.10% of dependents with COVID-19 had outpatient visits (assumption based on seasonal influenza data [34]); an assumed 3.57 employee workdays were missed per non-hospitalized COVID-19 episode; and 1.00% were hospitalized (based on Optum Clinformatics^®^ analysis), resulting in 9.10 employee workdays missed [42] (see Table S1 for seasonal influenza inputs).

Moderna COVID-19 mRNA Fall 2023/2024 vaccination was administered in September 2023, and outcomes were assessed over one year (until August 2024) in the model. The workplace Moderna COVID-19 mRNA Fall 2023/2024 vaccination program was assumed to prevent 23.5% and 57.3% of COVID-19 symptomatic infections and hospitalizations [43], respectively, across the season (see Supplemental File S1, Table S2b for details). Seasonal influenza vaccination was assumed to prevent 45.0% and 23.0% of symptomatic infections and hospitalizations, respectively, across the season, based on 2022–2023 data [34].

### 2.4. Scenario Analyses

Five scenario analyses were conducted to assess the impact on employer productivity losses and on the number of absent days using differing model assumptions reflecting factors relevant to employers (no coverage of dependents; long COVID impact, influenza hospitalization impact); to employees (caregiver scenario); and a conservative scenario. For this analysis, the model assumed a business with 10,000 employees. The impact was considered with 70% coverage of Moderna COVID-19 mRNA Fall 2023/2024 and seasonal influenza vaccination.

Scenario A took the ‘Employee only perspective’ i.e., not considering vaccination or burden of disease and costs for dependents. This scenario reflects companies which may not provide healthcare coverage to dependents of their employees. Scenario B was the ‘Cumulative long COVID’ scenario; assuming that long COVID patients would experience persistent symptoms, with an impact on productivity and medical costs beyond a one-year time horizon [44], thus leading to an accumulation and increased prevalence of long COVID over time due to more long COVID patients being in the company. This scenario approximates a disease burden perspective over a 5-year time horizon. With an assumed probability of developing long COVID of 2.2% for each symptomatic infection, the prevalence of long COVID was assumed to be 10.53% over five years. Scenario C considered ‘Flu hospitalization recovery’; assuming the same number of absent days during post-hospitalization recovery for seasonal influenza as for COVID-19 (COVID-19 assumed 33.4 days of productivity losses during recovery time), resulting in an overall 39.4 days of productivity losses for hospitalized seasonal influenza patients [37]. Scenario D was a ‘Conservative scenario’; only assuming lost productivity for employees and dependents with medical attendance. Scenario E, a ‘Caregiver scenario’, assumed 1.4 days of lost productivity to care for non-hospitalized dependents, based on estimates of caregiver time off for dependents with seasonal influenza [42], instead of assuming the same productivity loss for non-hospitalized dependents and employees.

### 2.5. Sensitivity Analyses

Comprehensive sensitivity analyses were conducted to assess the impact on employer productivity losses when varying key input parameters, informed by sensitivity analyses conducted in related COVID-19 vaccination cost-effectiveness analyses [24,45]. For this analysis, the model assumed a business with 10,000 employees. The impact on productivity was considered with 70% coverage of Moderna COVID-19 mRNA Fall 2023/2024 and seasonal influenza vaccination. The following key model probabilities were varied by plus or minus 20%: symptomatic infection; outpatient visit for symptomatic infection; hospitalization for symptomatic infection (employee and dependent); hospital death (employee and dependent); long COVID for symptomatic infection. In addition, the following key inputs were also varied by plus or minus 20%: vaccine effectiveness against hospitalization and against symptomatic infection; absent days for a non-hospitalized and hospitalized episode (employee and dependent); lost productivity cost per day; and replacement cost (for employees with long-term sick leave or who do not return to work).

## 3. Results

Without a COVID-19 workplace vaccination program in place, a business with 10,000 employees can expect to see 3869 annual symptomatic COVID-19 cases (with 31 hospitalizations) resulting in 18,175 absent days (of which 3771 are due to long COVID cases). For employers, this represents a productivity loss of USD 5.08 million with medical costs of USD 1.00 million (for self-insured employers). For employees, this represents USD 0.86 million in medical costs and USD 1.04 million in lost wages (from insufficient sick leave coverage) (Table 1). Without a seasonal influenza vaccination program, the disease impact is lower than for COVID-19 yet remains important. Employers can expect 1655 cases (and 10 hospitalizations), and 5333 absent days per year. For employers, this results in a lost productivity cost of USD 1.49 million and medical costs of USD 0.22 million from seasonal influenza; and for employees, the medical cost was estimated at USD 0.12 million (Table 1).

The results indicate that for a business with 10,000 employees, Moderna COVID-19 mRNA Fall 2023/2024 vaccination of employees and their dependents (with current coverage rates) could prevent on average 445 infections, 10 hospitalizations and 0.1 deaths due to COVID-19, and 1837 days of sick leave (of which 153 are due to long COVID). For the employer, this translates into savings of USD 183,346 in COVID-19 medical costs (for the self-insured employer) and savings of USD 513,544 in averted COVID-19 lost productivity costs. For employees, this represents savings of USD 135,519 in medical costs and savings of USD 48,809 in lost wages averted. By comparison, seasonal influenza vaccination of employees and their dependents (with current coverage rates) could prevent 325 infections, 1 hospitalization and 0.03 deaths due to seasonal influenza, and 1044 days of sick leave. Thus, saving the employer USD 30,964 in influenza-associated averted medical costs and USD 292,083 in influenza-associated averted lost productivity; while saving the employees USD 17,202 in averted medical costs (Table 1). No wage loss for seasonal influenza was observed as the number of days absence due to seasonal influenza for both a non-inpatient and an inpatient episode was less than 10 days.

**Figure 2:**
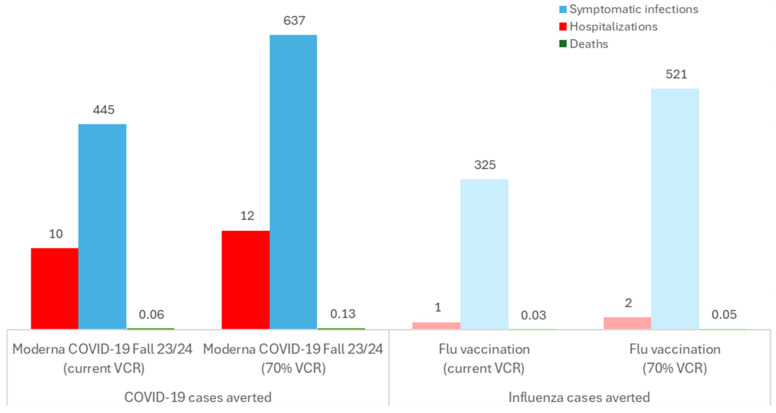
Disease impact averted with Moderna COVID-19 mRNA Fall 2023/2024 and seasonal influenza vaccination versus no vaccination (business N = 10,000 employees). COVID-19: Coronavirus disease 2019; Flu: seasonal influenza; Moderna COVID-19 Fall 23/24: Moderna COVID-19 mRNA Fall 2023/2024 vaccination; VCR: vaccination coverage rate. Note: a lighter shade is used for influenza outcomes.

Current vaccination coverage rates are suboptimal compared with seasonal influenza targets of 70% coverage [25]. Figure 2 illustrates how increasing Moderna COVID-19 mRNA Fall 2023/2024 and seasonal influenza vaccination coverage to 70% would further reduce the disease burden for employers and employees (e.g., 637 and 521 fewer COVID-19 and seasonal influenza cases, respectively, versus no workplace vaccination program), with fewer hospitalizations, deaths, and sick days as a result (Figure 2).

**Figure 3:**
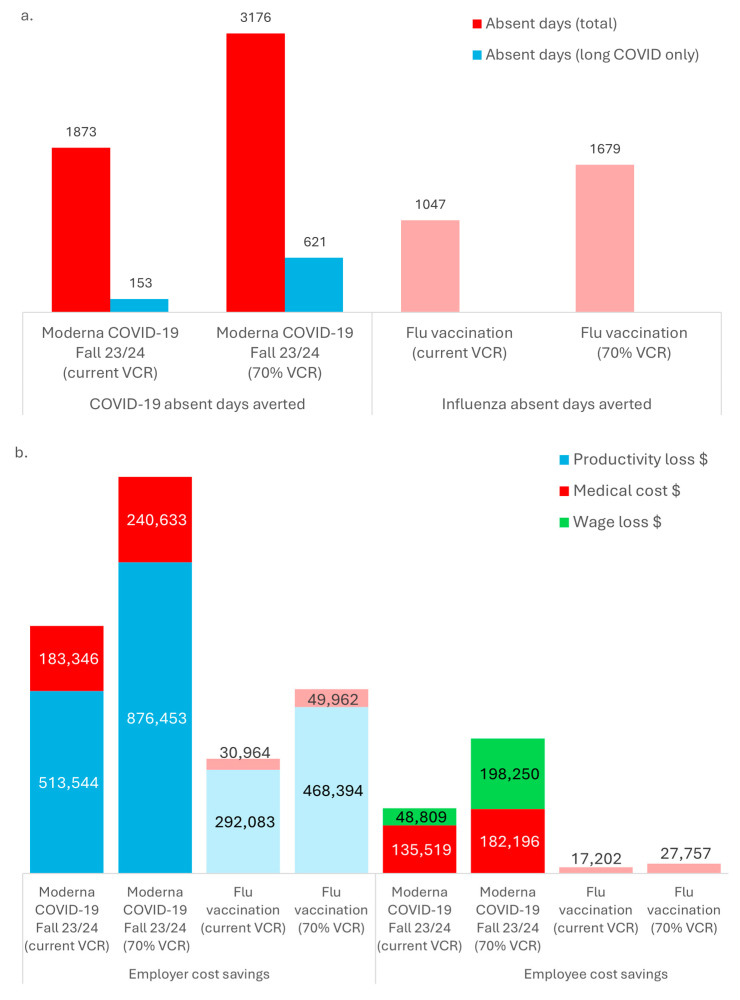
(**a**) Absent days averted; (**b**) Employer and employee cost savings—with Moderna COVID-19 mRNA Fall 2023/2024 and seasonal influenza vaccination versus no vaccination (N = 10,000 employees). Flu: seasonal influenza; M: million; Moderna COVID-19 Fall 23/24: Moderna COVID-19 mRNA Fall 2023/2024 vaccination; VCR: vaccination coverage rate. Note: a lighter shade is used for influenza outcomes.

Figure 3a shows the number of absent days averted following workplace vaccination for COVID-19 and seasonal influenza both at a current vaccination coverage rate as well as at a coverage rate of 70%. At 70% vaccination coverage, 3176 absent days due to COVID-19 (including 621 absent days due to long COVID) and 1679 absent days due to seasonal influenza are averted. Figure 3b illustrates the economic gains for employers and employees, of a Moderna COVID-19 mRNA Fall 2023/2024 and seasonal influenza workplace vaccination program at current vaccination coverage rate as well as at 70% coverage rates. For example, at a 70% vaccination coverage rate for COVID-19 alone, employers could save USD 876,453 in lost productivity costs averted and USD 240,633 in medical costs averted; and employees could save USD 182,196 in medical costs averted and USD 198,250 in lost wages averted. Thus, the increase in vaccination coverage rate from current VCR to 70% corresponds to additional cost savings of USD 362,909 in lost productivity costs and USD 57,287 in medical costs averted for employers as well as USD 46,677 in medical costs and USD 149,441 in lost wages averted. Cost savings were lower but still important for seasonal influenza cases prevented (e.g., for employers, savings of USD 49,962 for medical costs and USD 468,394 for lost productivity costs averted; for employees, savings of USD 27,757 for medical costs averted) (Table 1).

### 3.1. Scenario Analyses

**Figure 4:**
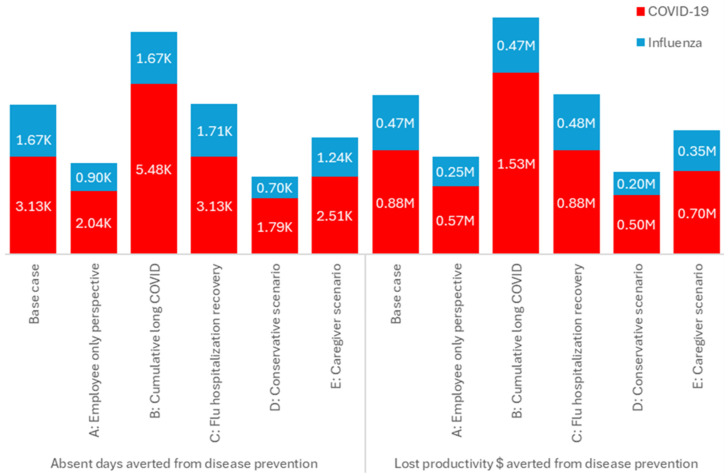
Vaccination impact: Impact of scenarios on absent days and lost productivity (USD) prevented by vaccination with 70% coverage Moderna COVID-19 mRNA Fall 2023/2024 and seasonal influenza workplace vaccination. Flu: seasonal influenza; K: thousand; M: million.

With a workplace vaccination program (at 70% coverage) in place, the largest benefits in absent days and lost productivity costs averted were seen in scenario B, assuming the cumulative impact from persistent long COVID than in the base case (i.e., resulting in 5482 absent days averted versus 3132 in the base case, and averted lost productivity costs of USD 1.53 million versus USD 0.88 million in the base case). Scenarios A, D, and E with their focus on employee impact only, or reduced impact in employees/dependents, had fewer absent days and lost productivity costs averted than the base case (Figure 4).

### 3.2. Sensitivity Analyses

**Figure 5:**
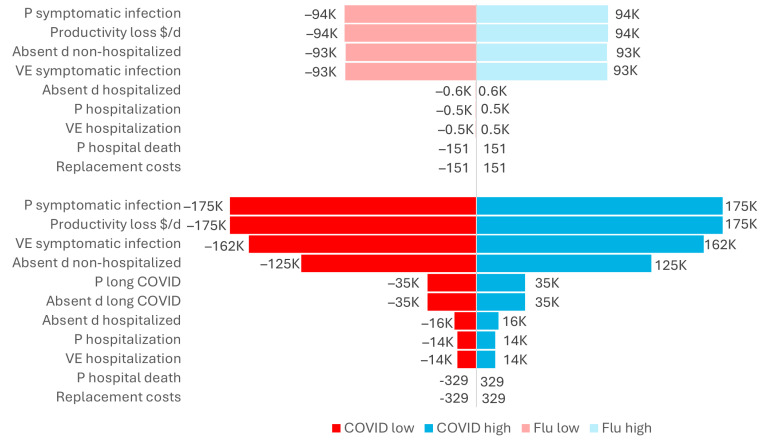
Vaccination impact: Tornado chart showing impact of varying inputs (+/−20%) on employer lost productivity (USD) prevented by vaccination, with 70% coverage Moderna COVID-19 mRNA Fall 2023/2024 and seasonal influenza workplace vaccination. d: days; flu: seasonal influenza; P: probability; VE: vaccine effectiveness.

With workplace vaccination (at 70% coverage) in place, the probability of symptomatic infection and the lost productivity cost per day had the largest impact on employer lost productivity (e.g., an increase of 20% in the probability of infection could increase employer costs saved by USD 175,000 in averted lost productivity). Varying the inputs for vaccine effectiveness against symptomatic infection and number of absent days for non-hospitalized employees also had a large impact on the overall costs of lost productivity prevented by vaccination (Figure 5).

## 4. Discussion

To inform implementation of COVID-19 workplace vaccination, this analysis estimated the workplace impact of COVID-19 disease for US employers and employees. With no COVID-19 workplace vaccination in place, for an average US business with 10,000 employees, there were an estimated 18,175 sick leave days per year due to COVID-19, with employer lost productivity costs of USD 5.08 million. Employee costs without COVID-19 workplace vaccination totaled USD 1.90 million in medical costs and lost wages.

Implementing COVID-19 workplace vaccination (with 70% vaccination coverage) prevented 3132 sick leave days due to COVID-19, saving employers USD 876,453 in COVID-19 related lost productivity and USD 240,633 in medical costs; while saving employees USD 182,196 in medical costs and USD 198,250 in lost wages. These results highlight the significant economic benefits for employers, as well as the substantial economic benefits for employees in addition to improving health, morale, and convenience for the employee [46]. By comparison, workplace seasonal influenza vaccination (with 70% vaccination coverage) prevented 1675 sick leave days, saving the employer around USD 468,394 in lost productivity costs and USD 49,962 in medical costs; and saving the employees around USD 27,757 in medical costs. Thus, increasing seasonal influenza coverage from current rates to the target of 70% could prevent an additional 630 absent days, and save the employer an additional USD 176,312 in lost productivity costs prevented.

In scenario analyses, considering the cumulative prevalence of long COVID had a large effect on overall absent days and lost productivity costs. This scenario highlights the potential enormous impact for employers when employees experience persistent long COVID symptoms and their subsequent effect on productivity. As the risk of developing long COVID symptoms increases with increasing number of reinfections [17,47], continued protection of employees against infection becomes even more important. In sensitivity analyses, the probability of having a symptomatic COVID-19 or seasonal influenza infection, as well as the cost per day of lost productivity, had the largest impact on the results, followed by vaccine effectiveness estimates against infection. Similar findings were reported for a workplace influenza vaccination study, showing that workplace program costs were lower when influenza was assumed to be more contagious and with higher vaccine effectiveness estimates [48].

The analysis found that the workplace burden and financial impact of COVID-19 was substantially higher than seasonal influenza, driven by higher incidence rates and the long COVID burden. Thus, COVID-19 vaccination provided employers approximately twice the savings in disease burden related productivity losses than seasonal influenza vaccination. The burden findings reflect the situation in the US in general, with CDC data (2023/2024 season) showing hospitalization rates per 100,000 population of 141.8 for COVID-19 [49] versus 82.4 for seasonal influenza [50]. In addition, a retrospective US claims analysis (2022/2023 season) reported that COVID-19 hospitalization rates were 5.6 times (in 18–49-year-olds) and 4.2 times (in 50–64-year-olds) higher compared with seasonal influenza, as were COVID-19 outpatient visit rates (5.6 times higher than for seasonal influenza) [51].

Current vaccination rates are suboptimal compared with the US target rate of 70% (for seasonal influenza [25]). Despite this, the analysis estimated that even at lower vaccination coverage rates (reflecting current rates in the population), both employers and employees had significant benefits from workplace vaccination. Workplace vaccination programs present an opportunity to increase coverage among working-age adults. US healthcare workers cited the most common reasons for receiving a COVID-19 vaccine in the workplace was that it was offered free of charge and that the workplace was an easy or convenient place to receive a vaccination [21].

Similar benefits of workplace vaccination were also reported in an economic analysis of workplace seasonal influenza vaccination for emergency medical services personnel, showing cost savings from avoided absenteeism (USD 20,745), avoided presenteeism (USD 7988), lost productivity (USD 10,303), and avoided medical costs of treating employees with influenza (USD 2454) [22]. The authors conclude that despite influenza vaccination being recommended for all healthcare workers (to protect employees and patients and prevent absenteeism), there is a lack of knowledge about the potential economic benefits for employers, which may inhibit implementation of more workplace vaccination programs [22]. This study used a cost-effectiveness model and analysis commonly used by health economists. Another study, using a dynamic transmission model, also reported influenza workplace vaccination benefits in Belgium, in terms of reduced disease burden and employee absenteeism, and an increase in cases averted and cost savings as vaccination coverage increased [23]. While both studies demonstrated the impact of vaccination for employers, the methods used may not be easily understandable by business employers, and may not provide relevant health and economic impact evidence for employers to make decisions about workplace programs.

Workplace vaccination is recommended by the CDC in the US [46]. Other countries, such as the United Kingdom (UK), also recommend workplace vaccination against COVID-19 as an effective way to protect employees and clients/patients, as well as employers who may suffer significant losses due to absenteeism, with recent guidance (January 2024) provided for employers on implementing such a program [52].

### Strengths and Limitations

The aim of this analysis was to estimate the burden of COVID-19 associated disease and the potential impact of workplace vaccination for an average US business, based on population averages in the absence of data to estimate a representative company. However, workplace benefits may differ by type of workplace [53]. For more accurate estimates, the model inputs should be adjusted to fit the specific employer’s settings, adjusting vaccination coverage rate, average wage, cost per missed day, number of paid sick days, insurance co-pay, and percent of insured employees as well as capturing workplace-specific vaccination costs, including potential productivity losses due to vaccine administration and adverse events. A simplified static model was used to provide employer/employee-perspective relevant outcomes for decision-making. However, the static model did not allow for more detailed outcomes to be assessed, such as the impact of employer or employee behavioral actions (e.g., increased social distancing in the workplace), or the effect of company size on risks of transmission. If changes in behavior following vaccination were to occur among employees or employers, potentially resulting in precautionary actions to further reduce exposure to COVID-19, the study findings and their significance may be limited. Similarly, the simplifying assumption that effects can be scaled linearly with company size may be a limitation, as non-linear effects exist. For example, the median attack rate for COVID-19 outbreaks was found to be 15 times higher in small businesses (<50 employees) compared with large businesses (≥250 employees) [54]. A key limitation of these findings is due to the uncertainty regarding COVID-19 incidence, corresponding health outcomes and associated productivity losses in the post-pandemic era, and to the uncertainty around the vaccine effectiveness against emerging variants, as the SARS-CoV-2 virus is constantly evolving [24]. Although the body of evidence is rapidly evolving, the long COVID disease burden and associated vaccination benefits may potentially be underestimated in this analysis. For instance, evidence is emerging to show that long COVID can also occur following asymptomatic SARS-CoV-2 infection [55]; however, only symptomatic cases were assumed to have a workplace impact. Also, the analysis did not account for the increased risk of developing long COVID symptoms following reinfection [17,47], or for any potential disability insurance payments [17] and dropping out of the workforce. In addition, only productivity losses due to absenteeism were considered for employers, as data on COVID-19-related presenteeism are limited. However, some evidence suggests COVID-19 may exacerbate presenteeism [56], thus further research is needed to characterize and quantify this impact. The model also relied on seasonal influenza data for some inputs where COVID-19 inputs were lacking. Key limitations also exist with regards to the uncertainty of input parameters for modelling the disease burden and impact of current seasonal influenza vaccination in the workplace, such as vaccine effectiveness estimates relying on 2022/2023 season estimates of currently available standard egg-based seasonal influenza vaccination. Also, for simplicity, the model conservatively assumed that seasonal influenza does not trigger or exacerbate underlying chronic conditions [27]. However, extensive scenario and sensitivity analyses conducted showed robust results with regards to disease burden and benefits of COVID-19 and seasonal influenza vaccination preventing disease-related health and productivity losses.

## 5. Conclusions

COVID-19 and seasonal influenza continue to pose significant threats to public health and the economy, leading to considerable absenteeism and lost productivity, and imposing a substantial financial burden on employers and employees. This study demonstrates that implementing COVID-19 and seasonal influenza workplace vaccination programs can substantially reduce absenteeism, productivity and wage losses, and medical costs for both employers and employees.

## Figures and Tables

**Table 1:** Disease and economic impact for employers and employees—data presented for N = 1 K, N = 10 K, and N = 100 K employees.

	No Moderna COVID-19 mRNA Fall 2023/2024	Moderna COVID-19 mRNA Fall 2023/2024 (Current VCR)		Moderna COVID-19 mRNA Fall 2023/2024 (70% VCR)		No Seasonal Influenza Vaccination	Seasonal Influenza Vaccination (Current VCR)		Seasonal Influenza Vaccination (70% VCR)	
Disease impact		Prevented	Total	Prevented	Total		Prevented	Total	Prevented	Total
Symptomatic infection, n	387	44	342	64	323	166	33	133	52	113
	3869	445	3424	637	3232	1655	325	1330	521	1134
	36,688	4445	32,243	6371	30,317	16,554	3252	13,302	5215	11,339
Hospitalization, n	3.1	1.0	2.1	1.2	1.8	1.0	0.1	0.9	0.2	0.8
	31	10	21	12	18	9.6	1.0	8.6	1.5	8.0
	306	100	206	123	183	96	9	86	15	80
Death, n	0.03	0.01	0.02	0.01	0.02	0.03	0.00	0.03	0.01	0.02
	0.33	0.06	0.27	0.13	0.20	0.33	0.03	0.30	0.05	0.28
	3.29	0.58	2.71	1.32	1.97	3.29	0.32	2.97	0.53	2.76
Economic impact		Prevented	Total	Prevented	Total		Prevented	Total	Prevented	Total
Absenteeism (total), d	1818	184	1634	313	1504	533	104	429	167	366
	18,175	1837	16,338	3132	15,043	5333	1044	4289	1674	3659
	181,752	18,374	163,379	31,324	150,429	53,333	10,442	42,891	16,744	36,589
Absenteeism (long COVID only), d	377	15	362	62	315	NA	NA	NA	NA	NA
	3771	153	3618	621	3150					
	37,706	1529	36,178	6209	31,497					
Productivity loss employer, USD	508,008	51,354	456,654	87,645	420,363	149,418	29,208	120,210	46,839	102,579
	5,080,084	513,544	4,566,540	876,453	4,203,632	1,494,180	292,083	1,202,097	468,394	1,025,785
	50,800,848	5,135,445	45,665,404	8,764,529	42,036,319	14,941,798	2,920,826	12,020,972	4,683,943	10,257,855
Medical cost employer, USD	100,025	18,335	81,691	24,063	75,962	21,859	3096	18,763	4996	16,863
	1,000,253	183,346	816,907	240,633	759,620	218,595	30,964	187,631	49,962	168,633
	10,002,530	1,833,462	8,169,068	2,406,327	7,596,204	2,185,949	309,643	1,876,307	499,619	1,686,330
Medical cost employee, USD	85,596	13,552	72,044	18,220	67,376	12,144	1720	10,424	2776	9369
	855,956	135,519	720,437	182,196	673,760	121,442	17,202	104,239	27,757	93,685
	8,559,557	1,355,188	7,204,369	1,821,958	6,737,599	1,214,416	172,024	1,042,393	277,566	936,850
Wage loss (unpaid sick leave) employee, USD	103,975	4881	99,094	19,825	84,150	0	0	0	0	0
	1,039,753	48,809	990,944	198,250	841,503	0	0	0	0	0
	10,397,527	488,092	9,909,435	1,982,499	8,415,028	0	0	0	0	0

USD: US dollar; d: days; n: number; VCR: vaccination coverage rate.

## Data Availability

The data presented in this study are available in this article and the Supplementary Materials.

## Supplementary Materials

The following supporting information can be downloaded at: https://www.mdpi.com/article/10.3390/jmahp13020017/s1, Table S1: Input parameters; File S1: Detailed model calculations (including Table S2 for vaccine effectiveness and Tables S3-4 for long COVID productivity loss).
