# Supplementary material for: Workplace Vaccination Against COVID-19 and Seasonal Influenza in the United States: A Modeling-Based Estimation of the Health and Economic Benefits for Employers and Employees
Source: J Mark Access Health Policy. 2025 Apr 24;13(2):17. doi: 10.3390/jmahp13020017 (PMC12101233; doi:10.3390/jmahp13020017)
Supplement: Supplementary file 1 [file jmahp-13-00017-s001.zip › jmahp-3396226-supplementary.pdf]

SUPPLEMENTAL MATERIAL

Workplace vaccination against COVID-19 and seasonal influenza in the United States: a modeling based estimation of the health and economic benefits for employers and employees

Table S1. Input parameters

Input parameter	COVID-19	Seasonal influenza
Employer inputs		
Employees	N=10,000 (base-case)<br>N=1,000<br>N=100,000	
Dependents per employee	0.86 [26] based on average n of <18yo in families	
Mean wage, annual	$61,441.60 [29] based on 220d*8hours	
% employees insured	60% [27] based on % of employees taking coverage at firms offering health benefits	
Employee medical insurance co-pay	25% (assumption)	
Paid sick days	10d (assumption)	
Working population (1000s)	18–29y: 35,327 [28]<br>30–39y: 33,303<br>40–49y: 30,484<br>50–64y: 40,403<br>65–74y: 7,971<br>75–84y: 1,847	
Current % vaccinated (employees & dependents)	6m–17y: 14.4% [30]<br>18–49y: 14.7%<br>50–64y: 23.5%<br>≥65y: 35.7%	6m–17y: 53.9% [18, 31]<br>18–49y: 37.5%<br>50–64y: 51.5%<br>≥65y: 73.8%
Target vaccination rate (employees & dependents)	70% for all ages<br>[23] based on national flu vaccination target	
Vaccine inputs		
VE against symptomatic infection<br>(see appendix S1 for detailed derivation for COVID-19 VE)	23.52% [42]	45.00% [33]
VE against hospitalization<br>(see appendix S1 for detailed derivation for COVID-19 VE)	57.25% [42]	23.00% [33]
Probabilities		

Symptomatic infection (annual, employees 18–64y)	20.8% [22] based on employee population weighted incidence	8.9% [32]
Outpatient visit (for employee infection)	39.2% [33] based on flu data	
Outpatient visit (for dependent infection)	56.1% [33] based on flu data	
Hospitalization (employee 18–64y)	0.61% [54]	0.74% [55]
Hospitalization (dependent)	1.00% [54]	0.39% [55]
Hospital mortality (employee)	2.07% [34]	4.5% [55]
Hospital mortality (dependent)	0.37% [34]	0.99% [55]
Severe long COVID among symptomatic infected (with time off work)	2.2%	NA
Productivity loss		
Symptomatic infection (employee, no hospital)	3.57d [22]	3.2d [41, 56]
Symptomatic infection (dependent, no hospital)	3.57d [22] (assumption)	3.2d [41, 56] (assumption)
Symptomatic infection (employee, inpatient)	42.31d [22, 36] (includes recovery time)	6d [41]
Symptomatic infection (dependent, inpatient)	9.1d [41] based on flu	9.1d [41] (employee absence for caregiving)
Severe long COVID, annual	82.4d [12, 37]	NA
Replacement duration (in case of death)	56d [40]	
Costs (inflated to 2023 price level [38] as needed)		
Workday missed	$279.28 [29]	
Replacement cost	$14,724 based on replacement duration* cost of workday missed	
Outpatient care	$477.17 [22] (assumed same for dependents)	$328.40 [38, 57]
Hospitalization	$22,853.01 [22] (assumed same for dependents)	$28,488.20 [38, 57]
Post-COVID follow-up cost over 1 year for any medically attended COVID-19 infection	$517.32 [38, 39] (assumed same for dependents)	NA

COVID-19: coronavirus disease 2019; D: day; flu: influenza; M: month; N: number; NA: not applicable; VE: vaccine effectiveness; y: year; yo: year-olds

Appendix File S1 – Detailed model calculations

Vaccine effectiveness calculations

COVID-19 incidence rates per month over the 1-year model horizon, for age groups 18–29 years, 30–39 years, 40–49 years and 50–64 years [22], take account of the seasonal pattern of COVID-19. These incidence rates were weighted by the population size of the age groups [57] (**Table S2a**), and used in the calculation of the average annual vaccine effectiveness (VE).

VE against infection and hospitalization was assumed to have a starting VE which declined each month linearly according to the waning rate of 4.75% for VE against infection and 1.37% for VE against hospitalization [22]. The starting VE was back-calculated, based on the observed VE against infection of 33.10% and against hospitalization of 60.10% at median follow-up time of 63 days [42]. In the model, VE is calculated mid-month, using these observed VE values after 63 days of vaccination and assuming VE peaks two weeks after vaccination and then starts to decline. The estimated annual VE, considering population-weighted incidence, was 23.52% against infection and 57.25% against hospitalization (**Table S2b**). These estimates provide an approximation, based on the assumption that individuals are vaccinated at the start of the season.

Table S2a. COVID-19 population-weighted [57] incidence rates (%) by month and age group based on [22]

Ages	M1	M2	M3	M4	M5	M6	M7	M8	M9	M10	M11	M12
18-29 years	0.49	0.67	0.80	0.89	0.81	0.60	0.47	0.32	0.23	0.16	0.13	0.11

30-39 years	0.42	0.57	0.68	0.77	0.70	0.52	0.41	0.27	0.20	0.14	0.11	0.09
40-49 years	0.37	0.51	0.61	0.69	0.64	0.48	0.37	0.25	0.18	0.12	0.10	0.08
50-64 years	0.45	0.63	0.77	0.90	0.85	0.65	0.51	0.34	0.24	0.16	0.13	0.11
**Total 18-64y**	**1.73**	**2.38**	**2.86**	**3.24**	**3.00**	**2.25**	**1.76**	**1.18**	**0.85**	**0.59**	**0.46**	**0.39**

Table S2b. Vaccine effectiveness per mid-month for ages 18–64 years

	**M1**	**M2**	**M3**	**M4**	**M5**	**M6**	**M7**	**M8**	**M9**	**M10**	**M11**	**M12**
VE against infection												
Linear (%)	40.23	35.48	30.73	28.35	23.60	18.85	14.10	9.35	4.60	0.00	0.00	0.00
Incidence-weighted (%)	3.37	4.07	4.25	4.44	3.42	2.05	1.20	0.53	0.19	0.00	0.00	0.00
**Cumulative incidence -weighted VE against infection over 1 year**												**23.52**
VE against hospitalization												
Linear (%)	62.16	60.79	59.42	58.73	57.36	55.99	54.62	53.25	51.88	50.51	49.14	47.77
Incidence-weighted (%)	5.20	6.98	8.22	9.19	8.32	6.10	4.64	3.04	2.12	1.43	1.10	0.91
**Cumulative incidence -weighted VE against hospitalization over 1 year**												**57.25**

Long COVID productivity loss calculations

Overall, 2.19% of symptomatic COVID-19 cases experienced persistent long COVID [22]. Of these, 33.28% stopped working [37] i.e., 0.73% overall stopped working; and the remaining 1.46% were assumed to keep working with an impact on productivity.

The overall productivity loss due to severe long COVID of 82.41 days was based on productivity losses in people working with absenteeism days; people who stopped work for a period of time and then returned; and people who stopped work for the model time horizon of 1 year. Proportions of people who returned to work or remained out of work within 0–2 months, 2–6 months, 6–12 months and >1 year after COVID-19 were based on the distribution from the New York State Insurance Fund (NYSIF) [12] (**Table S3**).

Productivity loss due to long COVID absenteeism was estimated at 5.19 days per month [22].

For those who kept working with long COVID, the productivity loss per month (5.19 days [22]) was multiplied by 12 months (i.e., total 62.28 days of lost productivity). For those who eventually returned to work, lost productivity accounted for fully absent days and days after returning to work but still experiencing absenteeism (i.e., total 97.32 days of lost productivity). For those who did not return to work, 62% were assumed to have been out for more than a year (i.e., total 179.20 days of lost productivity). The overall productivity loss due to severe long COVID, considering the three groups' average days lost, was 82.41 days (**Table S4**).

Table S3. Estimates of proportions returning to work and stopping work over time based on NYSIF report [12]

	% returned to work	% not returned to work	Number (%) returned	Number (%) did not return
**<2m**	95.83%	4.17%	373 (59.93%)	16 (5.77%)
**2–6m**	94.37%	5.63%	204 (32.74%)	12 (4.33%)
**6m–1y**	22.73%	77.27%	25 (4.07%)	86 (30.68%)
**≥1y**	10.87%	89.13%	20 (3.26%)	166 (59.20%)
			**622 (100%)**	**281 (100%)**

m: month; NYSIF: New York State Insurance Fund; y: year

Table S4. Calculations and assumptions for overall productivity loss for long COVID

	Calculations and assumptions	Days lost/y
Kept working with absenteeism	5.19d off/m *12m	62.28d
Stopped and returned to work	• 59.93% had 1m off + 11m with 5.19d off/m<br>• 32.74% had 4m off + 8m with 5.19d off/m<br>• 4.07% had 9m off + 3m with 5.19d off/m<br>• 3.26% had 12m off	97.32d
Stopped work	• Assumed 62% had 12m off	179.20d

	Among remaining 38%:<br>• 57.14% (16/28) stopped for 4m<br>• 42.86% (12/28) stopped for 9m	
**Average overall productivity loss for long COVID**	• 1.46% who kept working with 62.28d lost<br>Among 0.73% who stopped work:<br>• 68.91% (622/902) returned to work with 97.32d lost<br>• 31.09% (281/902) who did not return with 179.20d lost	**82.41d**

d: day; m: month; y: year
